# Selenium in Oncology: From Chemistry to Clinics †

**DOI:** 10.3390/molecules14103975

**Published:** 2009-10-12

**Authors:** Oliver Micke, Lutz Schomburg, Jens Buentzel, Klaus Kisters, Ralph Muecke

**Affiliations:** 1 Department of Radiotherapy and Radiation Oncology, Franziskus Hospital, Kiskerstraße 26, D-33615 Bielefeld, Germany; 2 Institute for Experimental Endocrinology, Charité Berlin, Germany; E-Mail: lutz.schomburg@charite.de (L.S.); 3 Department of Otolaryngology, Südharz Hospital Nordhausen, Germany; E-Mail: jens.buentzel@shk-ndh.de (J.B.); 4 Department of Internal Medicine, St. Anna Hospital, Herne, Germany; E-Mail: kisters@annahospital.de (K.K.); 5 Department of Radiotherapy, Lippe Hospital Lemgo, Germany; E-Mail: Ralph.Muecke@klinikum-lippe.de (R.M.)

**Keywords:** selenium, oncology, cancer prevention, cytoprotection, radioprotection, radical scavenger

## Abstract

The essential trace element selenium, which is a crucial cofactor in the most important endogenous antioxidative systems of the human body, is attracting more and more the attention of both laypersons and expert groups. The interest of oncologists mainly focuses in the following clinical aspects: radioprotection of normal tissues, radiosensitizing in malignant tumors, antiedematous effect, prognostic impact of selenium, and effects in primary and secondary cancer prevention. Selenium is a constituent of the small group of selenocysteine-containing selenoproteins and elicits important structural and enzymatic functions. Selenium deficiency has been linked to increased infection risk and adverse mood states. It has been shown to possess cancer-preventive and cytoprotective activities in both animal models and humans. It is well established that Se has a key role in redox regulation and antioxidant function, and hence in membrane integrity, energy metabolism and protection against DNA damage. Recent clinical trials have shown the importance of selenium in clinical oncology. Our own clinical study involving 48 patients suggest that selenium has a positive effect on radiation-associated secondary lymphedema in patients with limb edemas, as well as in the head and neck region, including endolaryngeal edema. Another randomized phase III study of our group was performed to examine the cytoprotective properties of selenium in radiation oncology. The aim was to evaluate whether sodium selenite is able to compensate a preexisting selenium deficiency and to prevent radiation induced diarrhea in adjuvant radiotherapy for pelvic gynecologic malignancies. Through this study, the significant benefits of sodium selenite supplementation with regards to selenium deficiency and radiotherapy induced diarrhea in patients with cervical and uterine cancer has been shown for the first time in a prospective randomized trial. Survival data imply that supplementation with selenium does not interfere with the positive biological effects of radiation treatment and might constitute a valuable adjuvant therapy option especially in marginally supplied individuals. More recently there were emerging concerns coming up from two large clinical prevention trials (NPC, SELECT), that selenium increases the possible risk of developing diabetes type II. Despite obvious flaws of both studies and good counterarguments, a controversial debate remains on the possible advantage and risks of selenium in cancer prevention. However, in the light of the recent clinical trials the potential benefits of selenium supplementation in tumor patients are undeniable, even if further research is needed.

## 1. Introduction

The essential trace element selenium, which is a crucial cofactor in the most important endogenous antioxidative systems of the human body, is attracting more and more the attention of laypersons and expert groups. The interest of oncologists mainly focuses in the following clinical aspects: radioprotection of normal tissues, radiosensitizing in malignant tumors, antiedematous effect, prognostic impact of selenium, and effects in primary and secondary cancer prevention [1].

## 2. Historical Perspective

August von Wassermann from the Rudolf Virchow Hospital in Berlin achieved in 1911 remissions of Ehrlich inoculation tumors in mice by intratumoral and intravenous injections of selenite [2]. von Öfele and Wolff (1912) successfully used sodium selenite as well as potassium cyanate in the treatment of gastric cancer [3]. Walker and Klein observed in 1915 the complete regression of smaller subcutaneous tumors after the oral application of subtoxic sodium selenite doses (1 mg per day). It is important to emphasize the theoretical approach of the physiological role of selenium, which the authors ascribe to the “hyperoxidation of the cells” - as they called it, by which they actually meant the consequences of the endogenic production of OH-radicals from H_2_O_2_ and hydroperoxides [4].

As early as 1920, a study by Watson-Williams was published in which hopeful results were reported of cancer patients treated with colloidal selenium [5]. Todd, who also used colloidal selenium, established significant survival figures with post-operative breast cancer patients [3]. In CML doses up to 200 mg selenocystine lead to decrease of leukocyte numbers and spleen size [6].

## 3. Biochemistry and Pathobiochemistry of Selenium

The essential trace element selenium is of fundamental importance to human health. As a constituent of the small group of selenocysteine-containing selenoproteins, selenium elicits important structural and enzymatic functions [7]. Selenium deficiency has been linked to increased infection risk and adverse mood states. Selenium has been shown to possess cancer-preventive and cytoprotective activities in both animal models and humans. It is well established that selenium has a key role in redox regulation and antioxidant function, and hence in membrane integrity, energy metabolism and protection against DNA damage. These and other functions are mediated through a small number of approximately 50 different selenoproteins encoded by 25 separate genes, which require adequate selenium availability for their regular biosynthesis and expression. Selenoproteins include several forms of the enzymes glutathione peroxidase (GPx), thioredoxin reductase and iodothyronine deiodinase [8].

Plasma selenium concentration is the most commonly used indicator of selenium status. Nutritional selenium intake, plasma selenium concentration and GPx activity display a positive correlation up to a threshold plasma selenium concentration (70–100 μg/L), beyond which the GPx activity plateaus [9]. This maximum GPx concentration is thought to represent repletion, and commensurate Selenium intake forms the basis for the recommended dietary requirement. Concentrations of other selenoproteins are also influenced by Selenium intake and may represent even better functional indicators of selenium status, but assay methods and reference standards are at an early stage, and comparisons between different studies are difficult [7].

There is a certain hierarchical expression of the selenoproteins, with relative preservation of the presumably more metabolically important members at lower intakes of selenium. Moreover, male and female organisms differ in their regulation of selenoprotein expression and epidemiological and intervention studies correlating health effects with the selenium status highlighted some important differences between the sexes [11]. Thus, we are far from understanding the health implications of suboptimal expression of the selenoproteins in humans [11,12,13].

### 3.1. In Vitro Cytoprotection by Selenium

Because of its antioxidant properties, selenium has traditionally been thought to possess protective capacities against the effects of radiation. Reactions of free radicals with sulphydryl groups from solute cysteine or peptides and proteins containing cysteine are thought to promote radiation protection [14]. The most powerful cysteine-containing natural antioxidant is glutathione. Artificial cytoprotectants like amifostine have been developed during recent years that also make use the binding of SH-groups to free radicals. The radioprotective effect of these drugs is supported by experimental as well as clinical data [15].

There are four characterized forms of GPx containing selenium [16]. Selenium glutathione peroxidases catalyze the elimination of hydrogen peroxide as well as organic peroxides (R–O–OH) by the oxidation of GSH. They contain a covalently bound selenium atom in the form of a selenocysteine molecule in their active center [17]. The substitution of selenocysteine with normal cysteine at the active site of GPx has been shown to dramatically reduce its enzymatic activity. The forms GPx-1 and GPx-2 are found in the cytosol, while GPx-3 is found in the plasma, and GPx-4 performs special functions in the metabolism of phospholipid hydroperoxides. Both overexpression and knockout models point to an important role of these enzymes in the protection against oxidative attacks [16].

Zhong *et al*. have shown that glioma cells possess antioxidant enzymes (superoxide dismutase, catalase) and that their sensitivity to glutathione-modifying drugs like BCNU is correlated to the catalase activity in these cells [18].

Mutlu-Türkoglu and coworkers demonstrated a protective effect of selenium and vitamin E on rat intestine that correlated with an increase of intestinal GPX activity [19]. These results seem to indicate a radioprotective effect of selenium on normal tissue. Hehr *et al*. showed a radioprotective effect of selenium in normal tissue (fibroblasts) but not in tumor cells [20]. Schleicher *et al*. found a stronger radioprotective effect in human endothelial cell lines than in cervix squamous carcinoma cells [21].

Gehrisch and Dörr [22] investigated the effects of systemic or topical administration of sodium selenite on early radiation effects (mucositis) in the mouse oral mucosa model. They found that the administration of sodium selenite during clinically relevant fractionated irradiation protocols has a significant effect during the initial treatment phase. Therefore, in clinical radiotherapy, the latent time to manifestation of confluent mucositis may be significantly prolonged, and hence the burden for the patient clearly reduced by selenium.

Margulies *et al*. [23], showed that radiation therapy differentially decreased cell number; with osteoblasts being shown to be the least sensitive to irradiation, the tumor cells had an intermediate sensitivity and monocytes were the most sensitive. Sodium selenite protected chondrocytes and osteoblasts from the negative effects of irradiation, while not protecting the tumor cells. It provided significant radioprotection to constituent bone cells while not protecting the tumor cells. Finally, selenium therapy provided an additional benefit beyond radioprotection by increasing cytotoxicity in nonirradiated and irradiated tumor cells. These experimental findings might be the basis for an improvement of the therapeutic ratio.

### 3.2. In Vitro Radiosensitization by Selenium

**Figure 1:**
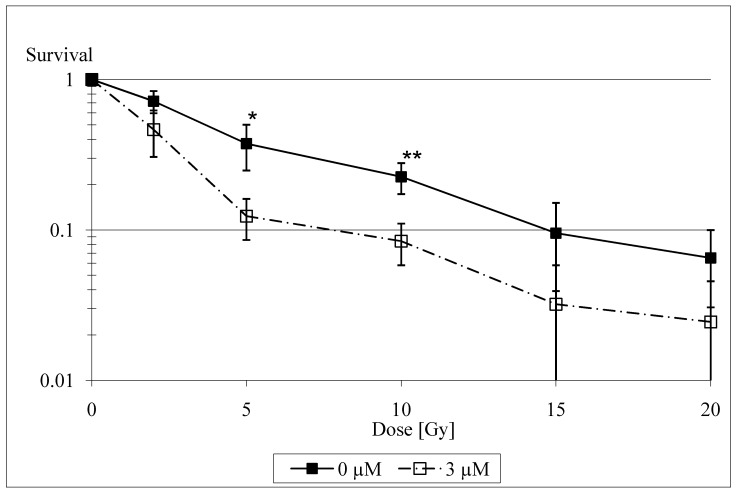
Logarithmic plot of cell survival at doses between 0 Gy and 20 Gy for selenite concentrations of 0 µM and 3 µM. Bars denote 95% confidence intervals. Asterisks denote significant values (* p < 0.05; ** p < 0.01).

There are several experimental data that illustrate the radiosensitizing capacities of selenium. Stewart *et al*. found that catalytically active selenium compounds (selenite and selenocystamine) can induce apoptosis and exert a cytotoxic effect by generating superoxide radicals [24]. The generation of superoxide is thought to be the main mechanism of selenium toxicity. It has been shown to be dose-related and limited to such compounds that react to form the selenite anion [25]. These results have been independently confirmed by Lanfear *et al*. [26], Davis [27] and Spallholz [25], and Lu *et al*. [28], who found selenite-induced DNA strand breaks and apoptosis and proposed various mechanisms for their coming about (oxidative stress by oxidation of glutathione, selenite-induced endonuclease activity). Frisk *et al*. [29] only investigated the influence of low doses of selenite on human glioma cells and thus did not find any influence of selenite on radiosensitivity. The recent study of our group [30] showed a radiosensitizing effect of selenite in irradiated rat glioma cells at medium, non-toxic concentrations of 2 to 3 µM, particularly at radiation doses >2 Gy (Figure 1).

If there really is a radiosensitizing effect of selenite on tumor cells at medium concentrations – as our results indicate – and at the same time a radioprotective effect on normal tissue – as the results of Mutlu-Türkoglu *et al.* [19] suggest, then selenite might be able to increase the therapeutic ratio for clinical radiotherapy. This hypothesis is also supported by the results from Hehr *et al*., who showed a radioprotective effect of selenium in normal tissue (fibroblasts) but not in tumor cells (squamous cell carcinoma) [20]. Moreover, Rodemann [31] described a pro-apoptotic effect.

On the other hand, the question of radiation sensitization or protection might also be a concentration issue: protection by its antioxidant properties at low concentrations versus sensitization by generation of superoxide at high, but non-toxic, concentrations [32].

## 4. Clinical Studies on Selenium

### 4.1. Clinical Studies on Cytoprotection by Selenium

Preliminary experimental and clinical evidence indicate that Se might function as an effective radio- and chemoprotector with the ability to alleviate side effects of tumor specific chemotherapy or radiotherapy treatments [20,21,22,33,34,35].

In 2003, an English retrospective study indicated a positive correlation between initial serum selenium levels and the dose delivery of chemotherapy and outcome (tumor response, long-term survival) in patients with aggressive non-Hodgkin´s lymphoma. The authors suggest that unlike most existing prognostic factors in aggressive non-Hodgkin’s lymphoma, selenium supplementation may offer a novel therapeutic strategy in this frequently curable malignancy [36]. In light of the results of a following experimental study the authors concluded that the selenium compounds methylseleninic acid and selenodiglutathione induce cell death in lymphoma cell lines and primary lymphoma cultures, which may be partly attributable to the generation of ROS [37].

Selenium deficiency is nearly the norm in cancer patients [38,39]. Radio- and chemotherapy as well as the suboptimal nutrition of cancer patients in the clinics might aggravate the situation in a selenium deficient patient even further, and increase the likelihood of radiation-induced side effects during and after therapy [34,35,39]. Supplementation of cancer patients with selenium at dosages of up to 2000 µg/day alone or in combination with vitamins has been reported in only a few studies as a way to improve quality of life [34,35,40].

**Figure 2:**
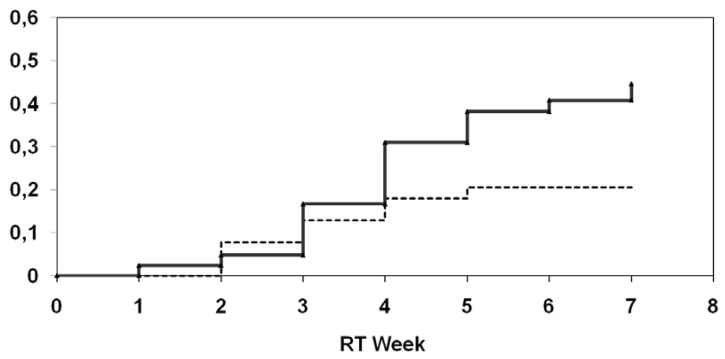
Univariate analysis (log-rank) for the incidence of at least diarrhea CTC 2 depending on supplementation of selenium (upper curve: without selenium; lower curve: with selenium).

Final results of a German multicenter randomized phase III study [41,42] provided more evidence for a beneficial effect of selenium supplementation in cancer patients undergoing radiotherapy: A total of 81 patients with gynaecologic cancers of the uterine cervix (n = 11) or endometrium (n = 70) after curative surgical treatment and a selenium deficiency (whole blood selenium less than 84 µg/l) were randomized before radiation therapy. In the supplementation group patients received 500 µg sodium selenite per os on the days of radiotherapy and 300 µg sodium selenite on the days without treatment till the last day of radiotherapy. In the control group adjuvant radiotherapy was given without supplementation of selenium. During treatment levels of whole blood selenium were measured after completing 50% of RT and at the end of RT using atomic absorption spectroscopy. Radiotherapy was delivered with a 6-18 MV linear accelerator, total doses ranged between 45 and 50 Gy (1.8-2.0 Gy single dose). Radiation-associated diarrhea was recorded weekly according to the CTC-criteria. Overall, 81 patients with a median age of 65 years (range 31-79) were randomized and evaluable. 39 were enrolled in the selenium group and 42 in the control group. Mean selenium levels did not differ between selenium group and control group upon study initiation, but were significantly higher in the selenium group at the end of RT. During supplementation, no selenium related side effects were observed. There was a statistically significant difference between the groups towards a lower incidence of diarrhea CTC 1-3 in week 4 in the selenium group compared to the control group (p = 0.01). The actuarial incidence of radiation-induced diarrhea of at least CTC 2 in the verum group was 20.5% compared to 44.5% in the control group (log-rank test: p = 0.04) (Figure 2).

**Figure 3:**
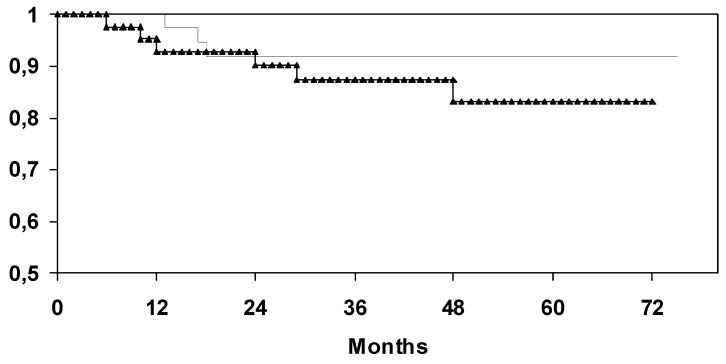
Univariate analysis (log-rank) for overall survival depending on supplementation of selenium (upper curve: with selenium; lower curve: without selenium).

By a median follow-up of 49 months (0-75), the actuarial 5-year disease-free survival rate of patients in the SG was calculated to be 80.1% compared to 83.2% in the CG (log-rank test: p = 0.74). By a median follow-up of 51 months (6-75), the actuarial 5-year overall survival rate of patients in the selenium group was calculated to be 91.9% compared to 83.1% in the CG (log-rank test: p = 0.34) (Figure 3).

For the first time, the significant benefit of sodium selenite supplementation with regard to selenium deficiency and RT-induced diarrhea in patients with cervical and uterine cancer has been shown in a prospective randomized trial. Disease free survival and overall survival data imply that supplementation with Se does not interfere with the positive biological effects of RT and might constitute a valuable adjuvant therapy option especially in marginally supplied individuals.

### 4.2. Antiedematous Effect of Selenium

A more recent interesting therapeutic option for lymphedema is selenium, the antiedematous effect of which is proven but not widely published or accepted. Early clinical studies have shown that oral selenium supplementation lowers oxygen radical production, causes a spontaneous reduction in lymphedema volume, increases the efficacy of physical therapy for lymphedema, and reduces the incidence of erysipelas infections in patients with chronic lymphedema at various sites. Unfortunately, these studies did not include large numbers of patients or use a standardized classification system for lymphedema, making objective evaluation of their outcomes difficult [43]. Kasseroller [44] reported promising results of a placebo-controlled, double blinded study of selenium in 179 postmastectomy patients suffering from secondary lymphedema. He described a significant reduction in edema volume, as well as improvement in skin-fold index in subjects with arm edemas. The incidence of erysipelas was also reduced in the selenium-treated group compared with the placebo group.

These results encouraged us to perform our own study using selenium for the treatment of radiation-induced lymphedema. The results of this exploratory study [40] involving 48 patients suggest that selenium has a positive effect on radiation-associated secondary lymphedema in patients with limb edemas as well as in the head and neck region, including endolaryngeal edema. The majority of patients showed a reduction in edema characteristics as classified by the Földi and Miller scoring systems. We also estimated that 65% of patients with interstitial grade III or IV endolaryngeal edema, who normally would require tracheotomy for treatment, could avoid surgical intervention. Although we could demonstrate a reduction in the circumferential difference between affected and unaffected sites in patients treated with selenium, as well as improvement in the skin-fold index and patient quality of life (as measured by a visual analog scale [VAS]), only the improvement in quality of life reached statistical significance. This is likely due to the small sample size.

The exact pharmacologic mechanisms are unknown. In the affected limbs of patients with chronic lymphedema, the production of free reactive oxygen radicals is enhanced as a result of lymphostasis, mechanical tissue compression, and chronic inflammation processes triggered by an excess of interstitial proteins and cellular debris. This promotes a variety of degenerative processes, worsening lymphostasis, and inflammation by tissue fibrosis. A reduction of free radicals caused by selenium-induced activation of GPx probably plays an important role in this pathological process [45,46]. Other preclinical studies have shown that selenium can protect human endothelial cells from oxidative damage by inducing GPx and thioredoxin reductase [47]. Analyzing these studies, it must be kept in mind that the histologic structure of lymphatic vessels and that of blood vessels are completely different. In particular, beyond a single layer of endothelial cells, lymphatic vessels have only a reticular fibrous network and no media consisting of smooth muscle cells [40].

### 4.3. Selenium in Cancer Prevention

Selenium was first associated with a reduced cancer risk in the late 1960s [48,49]. Larry C. Clark, from the Arizona Cancer Center, University of Arizona, Tucson, and colleagues studied the effectiveness of selenium supplementation for preventing development of new basal cell carcinoma (BBC) and squamous cell carcinoma (SCC) of the skin [50]. A total of 1,312 patients with previous skin cancers received placebo or 200 micrograms of the trace element selenium per day for a mean of 4.5 years and a total follow-up of 6.4 years. The patient population was recruited from the Eastern Coastal plain of the U.S., an area with relatively low selenium levels in soil and crops and also high rates of BBC and SCC.

The researchers found that selenium supplementation did not reduce the incidence of BBC or SCC. However, midway through the study, the authors also decided to evaluate the effect of selenium for preventing other types of cancers and for reducing cancer mortality. These secondary results indicate that when all cancers were studied, the selenium group had a 37 percent reduction in cancer incidence and a 50% reduction in cancer mortality, although there were no significant differences in deaths from all causes in the selenium group or the placebo group. Of the nearly 200 new cases of cancer diagnosed, the selenium group had 63 percent fewer prostate cancers, 58 percent fewer colorectal cancers and 46 percent fewer lung cancers than the placebo group. The authors concluded that these apparent beneficial effects of selenium supplementation require confirmation in independent trials of appropriate design [50].

A nonlinear association between serum selenium levels and all-cause and cancer mortality was published in 2008 based on the analysis of a representative sample of the US population with 13,887 adult participants. Increased serum Se levels were associated with decreased mortality up to concentrations of 130 μg/L. These results, however, raised the concern that higher serum selenium levels may be associated with increased mortality [51]. On the other hand, some studies revealed that higher selenium blood levels have no detrimental effect on human health. Karita *et al*. [52] reported on an high average serum selenium level of 146 µg/L among Tokyo residents without an increased mortality. Another study by Rajpathak [53] revealed that levels of toenail selenium are lower among diabetic men than among healthy controls.

In October 2008 the abrogation of the so called SELECT trial was announced, due to a number of reasons. In the study group of this prostate cancer prevention trial with selenomethionine supplementation, no positive effect on prostate cancer incidence was detected [54]. Moreover, a small and non-significant rise in the incidence of type 2 diabetes mellitus was observed.

Some potential issues need to be mentioned with respect to these surprising null results when supplementation attempts in the context of oncology are discussed: Firstly, the supplementation during the SELECT study was based on organic selenomethionine in contrast to selenium-enriched yeast as used before in the aforementioned and positive Clark study [50]. Here, a number of yeast-specific selenium-dependent compounds might have been missing, which could have an impact on the lack of chemopreventive activity observed. Moreover, selenomethionine-based supplementation trials are always very difficult to be interpreted since the supplement is incorporated unspecific into non-selenoenzymes due to its similarity to normal sulfur-methionine. The effect of selenomethionine on the expression of selenoenzymes can not be predicted or measured from patients’ blood samples. Therefore, it appears as if choosing sodium selenite as a supplement might represent a better alternative, for the effects of the supplementation can be verified by increased serum selenium or circulating selenoprotein concentrations. Secondly, the dosage management of an adjuvant long term selenium therapy should always be done according to the measured whole blood or serum selenium or selenoprotein values. The study population in the SELECT trial showed already a baseline serum selenium level of about 135 µg/L (corresponding to approx. 170 µg/L in whole blood and in accordance with published values [12]) and after supplementation reached abnormally high serum values of approx. 250 µg/L [54]. Finally, it must be taken into account that diabetes risk was not a primary study endpoint, and therefore, the trial was not stratified according to the common risk factors for diabetes type 2, e.g., body mass index etc [55,56]. Furthermore, the trial was rightly critisized for its 2×2 factorial design, which is inadequate for the study questions, and the low scientific evidence [56,57], on which the study conceptual design for tumorprevention and the choosen study medication and dosage was based [57,58]. In fact, an overall reduction of prostate cancer by 25% by a single agent appears much too optimistic. Concerning the supposed diabetic risk of selenium one must take into account, that the absolute increase of diabetes is just 0.7 % in the selenium only group (10% diabetes in the verum group versus 9.3%, in the placebo group) and did not reach statistical significance (p = 0.16) [57]. In addition, the data were not adjusted to the general increase of diabetes in the den US (between 2001 and 2007 about 16 %). Therefore, to our opinion this critirion cannot be appropriate to discontinue the study. All in all, the the SELECT trial is not adequate to prove either the infectivity of selenium to prevent prostate cancer or the hazard of selenium to trigger diabetes, and to discredit selenium for both [59]. The most important lesson we will learn from SELECT is that we must know much more details on the molecular and physiological mechnisms of selenium action in the human body [60,61].

So, whether surplus selenium intake of already well-supplied individuals confers any health benefit or rather leads to an increased risk for developing diabetes mellitus type 2 is currently an issue of concern and intensely controversial discussion [56,59,60,61].

Thus, we suggest measuring the selenium status in the tumor patients prior to, during therapy and in an aftercare situation to avoid side effects and optimize the odds for a positive Se supplementation effect in the clinics. There is currently no convincing rationale to administer Se to already well-supplied individuals [62]. Thirdly, selenium supplementation in the context of patients during radio- and chemotherapy represents a short-term focused intervention and not a chronic condition in contrast to the SELECT trial. Therefore, especially with respect to the marginally supplied patients who might have even aggravated their personal selenium deficiency further during the course of the illness, a selenium supplementation effort with the aim to increase the selenium status to optimal selenoprotein expression, i.e., into the range of 100 µg selenium/L or above appears advisable. This notion is in agreement with the high number of clinical studies correlating a reduced health risk and a better recovery from disease to the selenium status at the beginning or during the course of illness.

## 5. Conclusions

Selenium shows promising results concerning supplementation, cytoprotection and edema treatment in tumor patients. The role of selenium for cancer prevention in healthy well supplied individuals remains controversial, even though there appears to be a positive effect in the marginally supplied persons, if selenium is supplemented with the aim to increase the selenium status to optimal selenoprotein expression. However, in the light of recent clinical trials the potential benefits of selenium supplementation in tumor patients are undeniable. A tumor protective effect was not observed.
